# The Relationship between the Hamstring-to-Quadriceps Ratio and Jumping and Sprinting Abilities of Young Male Soccer Players

**DOI:** 10.3390/ijerph19127471

**Published:** 2022-06-18

**Authors:** Gürkan Diker, Artur Struzik, Sadi Ön, Raif Zileli

**Affiliations:** 1 Department of Physical Education and Sport, Faculty of Sports Science, Sivas Cumhuriyet University, Sivas 58000, Turkey; 2 Department of Biomechanics, Wroclaw University of Health and Sport Sciences, 51-684 Wrocław, Poland; 3 Department of Coaching Education, School of Physical Education and Sports, Ahi Evran University, Kırşehir 40100, Turkey; sadi.on@ahievran.edu.tr; 4 Department of Child Development, Faculty of Health Science, Bilecik Şeyh Edebali University, Bilecik 11000, Turkey; raif.zileli@bilecik.edu.tr

**Keywords:** CMJ, countermovement jump, H/Q ratio, isokinetics, knee joint, motor abilities, running, sport performance, squat jump, torque

## Abstract

The correct torque ratio between the knee joint extensor and flexor muscle groups can effectively prevent injuries to the anterior cruciate ligament and hamstring strain. However, it is unclear whether a high torque ratio of the knee joint flexor muscles to the extensor muscles is beneficial for sport performance. Therefore, the aim of the study was to investigate the relationship between the hamstring-to-quadriceps (H/Q) ratio and sprint times (10- and 30-m) and jump heights (CMJ—countermovement jump and SJ—squat jump) in soccer players. The study examined 26 young elite soccer players (age: 18.1 ± 0.7 years; body height: 1.77 ± 0.05 m; body mass: 72.7 ± 5.7 kg). Knee joint flexor and extensor peak torques were assessed using the Cybex dynamometer (at 60°/s, 120°/s and 180°/s). Additionally, each participant performed the CMJ, SJ, and 30 m sprint. A significant relationship was obtained between the H/Q ratio (60°/s) and 30 m sprint time (*r* = 0.47). The positive direction of this relationship may indicate an important role of knee joint extensors in sprinting performance. Moreover, the H/Q ratio was not significantly associated with the CMJ, SJ or 10 m sprint performance. The H/Q ratio should be considered together with the peak torque values in terms of the assessment of sprinting and jumping performance.

## 1. Introduction

Success in soccer is multifactorial and depends on physical, technical and tactical factors. Technical activities (such as the number of shots, successful passes, and winning one-on-one plays) can be better predictors of success in soccer than physical activities [1,2]. However, high tactical and technical levels are not possible without appropriate levels of individual physical (motor) abilities [1,2,3,4,5]. During a match, each soccer player performs 1000–1400 mainly short activities that change every 4 to 6 s [6]. Elite soccer players perform over 150 high-velocity running activities (at running velocities from 19.9 to 25.2 km/h) and over 40 sprinting activities (at running velocities over 25.2 km/h) [1,7]. High-intensity running occurs approximately every 70 s, whereas sprinting occurs approximately every 90 s, each lasting an average of 2–4 s [6]. Moreover, elite soccer players perform more high-intensity running and sprinting than moderate-level players [8,9]. Additionally, the number of high-velocity running activities and sprints increases from season to season [7]. High-intensity activities occur during the most important moments of a soccer match and contribute directly to winning possession of the ball or to scoring a goal [10].

Due to the substantial number of high-intensity short activities (such as accelerations, decelerations, cutting maneuvers, and changes in direction), the ability to reach and repeat high running velocity over a short distance is essential for high performance in soccer [6,10,11]. These activities are crucial to the outcome of the match and are associated with muscle size (mass), strength and power [12,13,14]. It is also known that players with greater muscle strength and power can reach greater running velocity and jumping height [12,14,15]. For example, Wisløff et al. [12] reported that maximum strength in half squats (1RM) determines 10 m (*r* = −0.94) and 30 m (*r* = −0.71) sprint times, and height (*r* = 0.68) of the countermovement jump with arm swing (CMJ—a vertical jump preceded by countermovement and arm swing) in high-level soccer players. Lehance et al. [16] noted significant relationships of knee joint extensor and flexor peak torques (measured at angular velocities of 60°/s) with squat jump (SJ—vertical jump from a steady squatting position) height (*r* = 0.45 and 0.48, respectively) and 10 m sprint time (*r* = −0.51 and −0.48, respectively) in professional and junior elite soccer players. Furthermore, muscle strength is one of the most important components in achieving high performance and preventing injuries in soccer players [17]. Pedersen et al. [18] reported that laboratory-based sprinting and jumping test performance showed an association with high-intensity physical match-play performance in high-level female football players.

While performing typical soccer tasks such as running, kicking and jumping, the concentric strength of the quadriceps should be balanced by eccentric hamstring strength [19]. Hamstring muscles help to prevent excessive quadricep contraction (by decelerating the extending knee joint), ensuring stability of the knee joint, protecting the ligaments and preventing tibial translation [19,20]. High absolute torque values of the knee joint extensors and flexors allow the player to execute kicking (striking), sprinting and jumping tasks during the match [21,22,23,24,25]. Knee joint extensor torque is a major determinant for performing the rapid knee extension during the swing phase while sprinting and during the take-off phase while jumping [25]. Furthermore, the knee extensor torque contributes to accelerating the center of mass of the body (COM) in a horizontal direction while sprinting and vertical direction while jumping and maintaining the height of the COM during the stance phase while sprinting [23,26]. On the other hand, knee joint flexors are important for joint stabilization [27]. Therefore, knee joint extensors and flexors are one of the most important muscle groups for sprinting and vertical jumping performance. However, in addition to the absolute values of knee joint torque, the ratio of the torque of the knee joint flexors to the extensors is also important. The commonly used “conventional” hamstring-to-quadriceps ratio (H/Q ratio) represents the ratio of concentric peak torque during knee flexion (mainly generated by the hamstring muscles) to concentric peak torque during knee extension (mainly generated by the quadriceps muscles) [28]. A low H/Q ratio (i.e., below the generally accepted threshold value of 60%) may lead to hamstring strain and/or anterior cruciate ligament (ACL) injury [19,29,30,31]. However, the relationships between the H/Q ratio and sports performance (e.g., sprinting and jumping) have not been sufficiently investigated [32,33]. Furthermore, it is not well known whether a high torque ratio of the knee joint flexor muscles to the extensors can improve the efficiency of sport performances.

Faude et al. [34] reported that 45% of goals are scored after straight sprints, 16% after jumps and 6% after sprints with a change in direction. Therefore, speed and jumping abilities are crucial in decisive match situations in soccer. Moreover, it was reported that a higher H/Q ratio is associated with longer professional training [35] and higher sports skills in soccer [27,36]. This may lead one to ask whether a higher H/Q ratio, which is favorable for preventing hamstring strain and ACL injuries, will also be beneficial for the performance of sprinting and jumping tasks. The relationships between the H/Q ratio and selected performance outputs (i.e., sprinting and jumping) are still not sufficiently explored in young soccer players. To our knowledge, no previous studies have examined the relationships between the conventional H/Q ratio and sprinting performance (U18 soccer players).

Therefore, the aim of the study is to investigate the relationships of the conventional H/Q ratio with sprint times (10- and 30-m) and jumps height (CMJ and SJ) in elite young soccer players. Additionally, based on previous reports [12,16,37,38,39], it can be expected that significant relationships should be found between speed abilities (10- and 30 m sprint times) and jumping abilities (CMJ and SJ heights), the two key abilities for scoring goals. Therefore, it is hypothesized that players who can jump higher should be characterized by shorter sprinting times.

## 2. Materials and Methods

### 2.1. Experimental Design

In this cross-sectional study, the participants attended laboratory testing in two sessions 48 h apart. During the first visit, anthropometric measurements and knee joint isokinetic muscle torque tests were performed at different angular velocities (i.e., 60°/s, 120°/s and 180°/s with a 5 min rest period in between). At the second visit, the CMJ, SJ, and 30 m sprint tests were performed twice, and further analysis was based on attempts with higher jump heights and shorter sprint times (for each participant). The participants were required to perform a standardized warm-up before the tests. To avoid variations in circadian rhythm, the tests were conducted during the same hours of the day (from 9.00 am to 2.00 pm, during the player’s regular training hours). The testing was completed in the regular season during the off week when no matches were played. All players participated in the tests after resting for 2 days after the last training. The participants were encouraged via verbal commands during the tests.

### 2.2. Participants

Twenty-six U18 category soccer players (age: 18.1 ± 0.7 years; body height: 1.77 ± 0.05 m; body mass: 72.7 ± 5.7 kg; body fat: 8.3 ± 2.1%) from a club competing in the Turkish Super League volunteered to participate in this study. Power analysis results for a *r*-Pearson coefficient showed that to detect an effect size of 0.55 at 80% power and alpha 0.05, a sample size of 23 was required. The players, who confirmed that they were in good health status and had no pain signs or recent injury in the lower limbs area, were examined. Failure to meet the above conditions excluded a person from participation. All players were licensed at the same club and had at least 5 to 6 years of soccer training experience. In addition, they regularly played 5 training sessions and one official match per week. The training sessions included tactics, technical exercises, small-sided games, high-intensity running, and strength training. The evaluation was conducted during the first half of the competitive season (November). Before the test, they were informed about the possible risks and benefits of the measurements and signed an informed consent form. The measurements were taken in the Physical Medicine and Rehabilitation laboratories at Ankara University Medical School.

### 2.3. Isokinetic Torque Measurements

A Cybex Norm 6000 isokinetic dynamometer (Cybex, Division of Lumex, Inc., Ronkonkoma, NY, USA) was used to measure the torque of the knee joint extensors and flexors at angular velocities of 60°/s, 120°/s and 180°/s (torque measurements accuracy within 1%). The back angle of the dynamometer chair was adjusted to 85°. The stabilizing connection point of the knee adapter was connected approximately 3 cm proximal to the dorsal surface of the foot on the extremity where the measurement was made. Before the test, the belts were fastened over the chest, pelvis, thighs, and other knee joint to maintain the body stabilization. In order to prevent the other knee from moving, the ankle was placed on the leg stabilizer, which was at the bottom of the chair. The torque value produced by the free leg in the 90° extension of the knee joint was determined with a dynamometer in order to eliminate the effect of gravity in advance of the measurements. In the outset of the test, the subject was asked to apply maximal knee flexion/extension torque to pass the test positively and to obtain the best results. The participant was seated on the dynamometer chair and performed five repetitions of maximal knee joint extension and flexion at each angular velocity for the right and left lower limb separately. Participants’ knee joint extension and flexion torque measurements were performed at the angular velocities of 60°/s (4 familiarization repetitions, then 15 s rest and then 5 main test repetitions), 120°/s (4 familiarization repetitions, then 15 s rest and then 5 main test repetitions) and 180°/s (4 familiarization repetitions, then 15 s rest and then 5 main test repetitions), respectively, in the fixed protocol of the dynamometer. The measurements were performed in a random order with additional verbal motivation, and the angular velocities were similar to those used in previous reports [19,40,41]. Further analysis was based on trials with the highest extensor and flexor peak torques (for each participant and for each angular velocity). The H/Q ratio was calculated using the following equation:

H/Q ratio = [(*F_l_* + *F_r_*)/(*E_l_* + *E_r_*)] · 100%, (1)

where (*F_l_* + *F_r_*) and (*E_l_* + *E_r_*) are the sums of the left and right lower limb peak torque values of knee joint flexors and extensors, respectively [32]. The calculation of the H/Q ratio using torque values of both lower limbs was deliberate because both lower limbs are used (simultaneously or cyclically) during the sprinting and jumping tasks.

### 2.4. Jumping and Sprinting Performance

Prior to the tests, a 10 min warm-up, which included jogging, a series of hops, and familiarization with tests, was administered. A Newtest contact mat (Newtest 1000, OY, Oulu, Finland) was used for the CMJ and SJ tests (flight time measurements accuracy ± 0.001 s and ± 2.0 mm of the jump height). The participants performed the CMJ by flexing their lower limbs (countermovement) and immediately performing a vertical jump. During the SJ, the participants flexed their knee to 90°, and after sustaining this position for 3 s, they jumped vertically. The participants completed two repetitions of the CMJ and the SJ as high as they could with their arms akimbo [42]. The jump was repeated if the lower limbs were flexed during the flight phase. A 1 min rest interval was given between each trial. Further analysis was based on higher jumps for each type of each participant. The jump height (*h*) was calculated based on the duration of the flight phase (*t_f_*):

*h* = [*g* · (*t_f_*)^2^]/8, (2)

where *g* is the acceleration due to gravity.

The participants performed the 30 m sprint twice. The task to be performed was to cover the designated distance (30 m) in the shortest possible time. Following the warm-up, all participants completed the 30 m maximum sprint within the marked 1 m area. The subject started from a standing position placing his forward foot just behind the starting line according to their natural starting position. When the photocell at the starting line was passed, the time measurement was automatically started. The participant decided himself when to start the sprint. Passive recovery between sprints was 3 min. The 10- and 30 m split times were obtained using a photocell system (Newtest 1000, OY, Oulu, Finland). Photocells were positioned at the starting line, 10 m and 30 m, at a height of 1 m (time measurements accuracy ± 0.001 s). The test was performed on a grass pitch (temperature: 11 °C, relative humidity: 27.5%, weather: very cloudy, time of the day: 9.00 am–12.00 pm). The shorter times for each participant (10- and 30-m) were taken for further analysis [43].

### 2.5. Statistical Analysis

IBM SPSS software (version 24.0, IBM Corp., Armonk, NY, USA) was used for statistical analysis. The distribution of data were checked using the Shapiro–Wilk (*W*) test. There were no statistically significant differences for all variables (*p* > 0.05). Therefore, parametric tests were used for further analyses. Descriptive statistics are reported as the mean ± standard deviation (SD) and coefficient of variation (CV). The relationships between the H/Q ratio, knee joint extensors peak torque, knee joint flexors peak torque and the CMJ height, SJ height, 10 m split time, and 30 m sprint time were evaluated using *r*-Pearson’s correlation coefficient. Additionally, the relationships between speed abilities (10 m and 30 m sprint times) and jumping abilities (CMJ and SJ heights) were also evaluated. The usual scale for correlation coefficients was used for interpretation of *r* values: 0.0–0.1, trivial; 0.1–0.3, small; 0.3–0.5, moderate; 0.5–0.7, large; 0.7–0.9, very large; and 0.9–1.0 nearly perfect [44]. In this study, the following CV ranges were adopted for interpretation of variability: CV < 10, low; 10–20, average; 20–30, high; and CV > 30 not acceptable [45,46]. Additionally, a 95% confidence interval (CI) was calculated for each value of the correlation coefficient obtained. Statistical significance was set at *p* < 0.05.

## 3. Results

Table 1 contains the mean (±SD) and CV values of the H/Q ratio, peak torque of knee joint extensors and flexors, and variables that describe sprinting and jumping abilities.

On the basis of the CV value, it can be assumed that the sprint times, jump heights, and 60°/s H/Q ratio had a low variability and the other variables from Table 1 (knee joint extensor peak torque, knee joint flexor peak torque, 120°/s H/Q ratio, and 180°/s H/Q ratio) had an average variability. The H/Q ratio at an angular velocity of 60°/s was significantly correlated with 30 m sprint performance (*r* = 0.47, *p* < 0.05), while the H/Q ratio, knee joint extensors peak torque, and knee joint flexors peak torque at any angular velocity were not significantly associated with CMJ, SJ or 10 m sprint performance (Table 2). Additionally, no significant relationships were observed between sprint times and jump heights (Table 3).

## 4. Discussion

This study examined the relationships of the conventional H/Q ratio with sprint times (10- and 30-m) and jumps height (CMJ and SJ) in elite young soccer players. Some previous reports [16,32,47] have shown that the H/Q ratio is associated with CMJ but not SJ performance. However, this study found no significant relationships between the height of the jumps (CMJ and SJ) and the H/Q ratio at any angular velocities (60°/s, 120°/s and 180°/s).

Struzik and Pietraszewski [32] evaluated peak torques of knee joint extensors and flexors under isometric and isokinetic conditions in a group of female soccer players to identify the relationships between the conventional H/Q ratio and jumping variables. They reported that the H/Q ratio measured at some angular velocities correlated positively (moderate or large) with the heights of CMJ (at isometric, 30°/s, 60°/s, 90°/s and 120°/s) and drop jumps (DJ—a vertical jump after landing from a specific height) from 15 cm (at 60°/s, 90°/s and 120°/s) and 30 cm (at 120°/s) platforms, whereas the heights of DJs from 45 cm and 60 cm platforms showed trivial relationships with the H/Q ratio [32]. Moreover, positive relationships between CMJ take-off power and the H/Q ratio were reported by Struzik and Pietraszewski [32] at angular velocities of 60°/s, 90°/s and 120°/s and by Trzaskoma [47] under isometric conditions. Therefore, the lack of a relationship between the H/Q ratio and CMJ height in this study is unexpected. The CMJ is a complex movement in the stretch-shortening cycle, which involves eccentric-concentric muscle work in the countermovement and take-off phases. Therefore, the relatively high torque value of the knee flexors compared to the extensors (i.e., high H/Q ratio value) should positively affect the vertical jump height. The studied soccer players obtained H/Q ratios above 60% at all angular velocities (60°/s, 120°/s and 180°/s). However, it should be noted that a high H/Q ratio may occur with the relative weakness of both extensors and flexors of the knee joint, which may also explain the lack of relationship between the H/Q ratio and CMJ performance [33]. Indeed, the participants of this study achieved lower knee joint torque values (Table 1) than other groups of soccer players [16,17,23,25,27,35,48,49]. Therefore, relatively low values of knee joint extensor and flexor torques may explain the lack of a significant relationship between the H/Q ratio, knee joint extensors peak torque, knee joint flexors peak torque and CMJ height (Table 2).

Similar to our findings, Lehance et al. [16] reported that the H/Q ratio (at angular velocities of 60°/s and 240°/s) has a trivial relationship with SJ height. However, the SJ is not a movement in stretch-shortening cycle muscle action but only a concentric lower limb activity of extensor muscles during take-off. Therefore, the lack of a relationship between the H/Q ratio and SJ height was perhaps predictable. On the other hand, Tsiokanos et al. [22] showed that SJ height is related to knee joint extensor peak torque at angular velocities of 60°/s (*r* = 0.49), 120°/s (*r* = 0.57), and 180°/s (*r* = 0.59). Dauty et al. [50] also reported that knee joint extensor peak torque at 180°/s is associated with SJ (*r* = 0.51) and CMJ (*r* = 0.65) performance. Therefore, the peak torque of the knee joint extensors evaluated at a relatively high angular velocity is a better indicator of SJ performance than the H/Q ratio. However, no significant relationships between knee joint extensor peak torque and SJ height were found in this study (Table 2). The lack of the aforementioned relationships may confirm the fact that the obtained knee joint extensor peak torque values are relatively small.

No previous studies have examined the relationship between the conventional H/Q ratio and sprinting performance. During typical soccer tasks, such as (repeated) short distance sprinting, concentric knee joint extensor torque is balanced by the eccentric action of the knee joint flexors. Previous studies have shown associations between sprint performance and eccentric hamstring strength [51], 30 m sprint velocity and knee joint extensor peak torque [48], 30 m sprint performance and knee joint flexor rate of torque development [52] and the metabolic cost of running with a functional H/Q ratio [53]. In this study, a moderate relationship (*r* = 0.47) was obtained between the 30 m sprint time and H/Q ratio at 60°/s. However, this relationship is positive, so an increase in the H/Q ratio is accompanied by a longer running time. Such a relationship may suggest a weakness of the knee joint extensor muscle group and point to the important role of knee joint extensors in sprint performance [54]. This may also explain the lack of significant relationships between the H/Q ratio and 10 m sprint time (where lower limb power is essential) [37]. Cotte and Chatard [48] reported a negative relationship between knee joint extensor peak torques (at 180°/s, 240°/s and 300°/s) and 20–30 m sprint times, but not 0-10 or 10-20 m sprint times.

No significant relationships were observed between sprint times (10- and 30-m) and jump heights (CMJ and SJ) in the study group of soccer players. In many previous studies, significant negative relationships were found between jumping and sprinting performances [16,38,39]. Vescovi and McGuigan [55] noted that the relationships between CMJ height and linear sprint times were stronger at longer distances (27.4 and 36.6 m) than at shorter distances (9.1 and 18.3 m). Moreover, Boone et al. [56] reported that relationships between sprinting and jumping kinetics are limited. Wisløff et al. [12] reported relationships of CMJ height with both 10 m (*r* = 0.72) and 30 m sprint time (*r* = 0.60) in international male soccer players. However, the relationships obtained by Wisløff et al. [12] were positive, i.e., a higher jump height was accompanied by a longer running time (or vice versa). This may indicate a narrow specialization of players who preferred playing head or to use their speed. The above suggestion may also explain the lack of significant relationships between speed abilities (sprint times) and jumping abilities (jumps height) in the study group.

The limitations of this study include incomplete information about the applied training loads, gender (different results can be obtained in female groups) and sport (athletes practicing other sports or non-training individuals may have different levels and proportions of motor abilities). The age of the participants may also be a limitation of this study. The study group of soccer players was relatively young (U18), which may explain the relatively low knee joint torque values. At this age, soccer players have not yet reached their maximum level of motor abilities. However, the magnitude of muscle torque also depends on the sports skill level [35,36,57]. Relatively low values of knee joint torques may also result from general training, which was mainly carried out without a strong focus on lower limb strength. Additionally, young players may not yet be strictly assigned to specific playing field positions. The specific position of the players on the field implies not only particular morphotypes but also specific isokinetic torque profiles [58,59,60]. Therefore, for future research, it is advisable to conduct similar measurements on groups of adult soccer players and those practicing other sports.

The similar knee joint torque values (for extensors and flexors) obtained independently of different angular velocities (60°/s, 120°/s and 180°/s) should also be considered. The negative relationship between angular velocity and knee joint torque should be expected. However, some players may provide the “classically” shaped torque-angular_velocity curve, where torque decreases markedly with angular velocity increments, while other players may obtain a relatively “flat” torque-angular_velocity relationship with only small and insignificant torque decrements [61]. The above-mentioned factors such as age, training, sport, and low torque values may be responsible for similar torque values despite different angular velocities in studied group (especially for the left lower limb). The shape of the torque-angular_velocity curve probably depends on the ability to generate power output. The training necessary to reach the professional level creates a dominant lower limb in soccer players [62]. Despite this fact, soccer players can obtain unexpected results, e.g., higher knee joint extensor torque values for the non-dominant rather than dominant lower limb at certain angular velocities [63].

Another limitation that may have influenced the obtained relationships of the H/Q ratio with jumping and sprinting abilities is the lack of increase in the conventional H/Q ratio along with increasing angular velocity. The H/Q ratio values were almost the same for each angular velocity (60°/s, 120°/s and 180°/s). An increase in the conventional H/Q ratio has mainly been observed with an increase in angular velocity for male subjects [40,64]. The absence of this dependence in the studied group may also result from the relatively low knee joint extensor and flexor torque values. It should also be considered that the H/Q indicator has limitations. For example, peak torque values of the extensors and flexors occur at different knee joint flexion angles [64]. Hence, many authors are considering refining this predictor or providing alternative variants (e.g., functional H/Q ratio) so that the disproportions between antagonistic muscle groups can be better assessed [65,66,67,68].

## 5. Conclusions

To maximize running and jumping performance, a correspondingly high peak torque value of the knee joint extensors is required [69]. The positive relationship between the H/Q ratio and 30 m sprint time may suggest the important role of knee joint extensors in sprint performance. The results in this paper do not allow for a strong statement on this matter, as only in 22% of participants (*r* = 0.47) the variation in 30 m sprint time can be explained by the positive linear relationship between the H/Q ratio (at 60°/s) and 30 m sprint time. Moreover, the H/Q ratio was not significantly associated with the CMJ, SJ or 10 m sprint performance. Due to its limitations, H/Q ratio analyzed individually may have limited application. Therefore, the H/Q ratio should be considered together with the peak torque values of knee joint flexor and extensor in terms of use for the assessment of sprinting and jumping performance.

## Figures and Tables

**Table 1:** Mean ± SD and CV values of knee joint extensor and flexor peak torques, the H/Q ratio, jump heights and sprint times.

Knee Joint Torque Extensors				
	Left Lower Limb		Right Lower Limb	
Angular Velocity	mean ± SD (Nm)	CV (%)	mean ± SD (Nm)	CV (%)
60°/s	151.2 ± 20.9	13.8	164.7 ± 20.2	12.3
120°/s	156.3 ± 21,2	13.6	159.6 ± 23.3	14.6
180°/s	151.1 ± 20.9	13.8	158.7 ± 21.9	13.8
**Knee Joint Torque Flexors**				
	**Left Lower Limb**		**Right Lower Limb**	
	**mean ± SD (Nm)**	**CV (%)**	**mean ± SD (Nm)**	**CV (%)**
60°/s	98.9 ± 17.3	17.5	112.3 ± 12.5	11.1
120°/s	100.3 ± 16.3	16.3	110.1 ± 17.0	15.4
180°/s	97.5 ± 20.7	21.2	110.3 ± 18.4	16.7
**H/Q Ratio**	**mean ± SD (%)**		**CV (%)**	
60°/s	67.0 ± 5.4		7.4	
120°/s	66.9 ± 7.4		10.8	
180°/s	67.2 ± 9.0		13.2	
**Jump Heights**	**mean ± SD (m)**		**CV (%)**	
CMJ	0.363 ± 0.035		9.4	
SJ	0.329 ± 0.025		7.4	
**Sprint Times**	**mean ± SD (s)**		**CV (%)**	
10-m	1.63 ± 0.04		2.3	
30-m	4.03 ± 0.10		2.5	

**Table 2:** Values of the correlation coefficients (*r*) with 95% CI between knee joint extensors peak torque (right + left lower limb), knee joint flexors peak torque (right + left lower limb), H/Q ratio and CMJ height, SJ height, 10 m split time, and 30 m sprint time.

*r* (Lower, Upper 95% CI)	60°/s			120°/s			180°/s		
	Extensors	Flexors	H/Q	Extensors	Flexors	H/Q	Extensors	Flexors	H/Q
CMJ height	0.076	0.067	0.010	−0.012	0.048	0.090	−0.185	0.033	0.202
	(−0.32, 0.45)	(−0.33, 0.44)	(−0.38, 0.40)	(−0.40, 0.38)	(−0.35, 0.43)	(−0.31, 0.46)	(−0.53, 0.22)	(−0.36, 0.42)	(−0.20, 0.55)
SJ height	0.019	−0.202	−0.334	−0.058	−0.154	−0.380	−0.318	−0.318	−0.241
	(−0.37, 0.40)	(−0.55, 0.20)	(−0.64, 0.06)	(−0.44, 0.34)	(−0.51, 0.25)	(−0.67, 0.01)	(−0.63, 0.08)	(−0.63, 0.08)	(−0.58, 0.16)
10 m split time	−0.091	0.161	0.388	−0.065	0.124	0.199	−0.010	0.227	0.278
	(−0.46, 0.31)	(−0.24, 0.52)	(0.01, 0.67)	(−0.44, 0.33)	(−0.28, 0.49)	(−0.20, 0.54)	(−0.40, 0.38)	(−0.18, 0.57)	(−0.12, 0.60)
30 m sprint time	−0.292	−0.047	0.467 *	−0.097	0.066	0.322	0.058	0.117	0.323
	(−0.61, 0.11)	(−0.43, 0.35)	(0.10, 0.72)	(−0.47, 0.30)	(−0.33, 0.44)	(−0.08, 0.63)	(−0.34, 0.44)	(−0.28, 0.48)	(−0.07, 0.63)

* significant at *p* < 0.05.

**Table 3:** Values of the correlation coefficients (*r*) with 95% CI between speed abilities (10 m and 30 m sprint times) and jumping abilities (CMJ and SJ heights).

*r* (Lower, Upper 95% CI)	10 m Split Time	30 m Sprint Time
CMJ height	−0.191 (−0.54, 0.21)	−0.121 (−0.49, 0.28)
SJ height	−0.355 (−0.65, 0.38)	−0.358 (−0.66, 0.34)

## Data Availability

The data used to support the findings of this study are available from the corresponding author upon request.
